# A human immune data-informed vaccine concept elicits strong and broad T-cell specificities associated with HIV-1 control in mice and macaques

**DOI:** 10.1186/s12967-015-0392-5

**Published:** 2015-02-15

**Authors:** Beatriz Mothe, Xintao Hu, Anuska Llano, Margherita Rosati, Alex Olvera, Viraj Kulkarni, Antonio Valentin, Candido Alicea, Guy R Pilkington, Niranjan Y Sardesai, Muntsa Rocafort, Manel Crespo, Jorge Carrillo, Andrés Marco, James I Mullins, Lucy Dorrell, Tomáš Hanke, Bonaventura Clotet, George N Pavlakis, Barbara K Felber, Christian Brander

**Affiliations:** IrsiCaixa AIDS Research Institute - HIVACAT, Hospital Germans Trias i Pujol, Crta Canyet s/n., 08916 Badalona, Barcelona Spain; ‘Lluita contra la Sida’ Foundation, Hospital Germans Trias i Pujol, Badalona, Barcelona Spain; Universitat de Vic-Universitat Central de Catalunya (UVic-UCC), Vic, Spain; Human Retrovirus Pathogenesis Section, National Cancer Institute-Frederick, Frederick, MD USA; Human Retrovirus Section, National Cancer Institute-Frederick, Frederick, MD USA; Inovio Pharmaceuticals Inc, Blue Bell, PA USA; HIV Unit, Hospital de la Vall d’Hebrón, Barcelona, Spain; Centres Penitenciaris BCN, Barcelona, Spain; University of Washington, Seattle, WA USA; Nuffield Department of Medicine, University of Oxford, The John Radcliffe Hospital, Oxford, UK; The Jenner Institute, University of Oxford, Oxford, UK; Universitat Autònoma de Barcelona, Barcelona, Spain; Institució Catalana de Recerca i Estudis Avançats (ICREA), Barcelona, Spain

**Keywords:** HIV-1 T-cell immunogen, HIV-1 specific CTL, HLA, Immunogenicity, Subdominant, Viral fitness, CTL escape, T-helper epitope, Population coverage

## Abstract

**Background:**

None of the HIV T-cell vaccine candidates that have reached advanced clinical testing have been able to induce protective T cell immunity. A major reason for these failures may have been suboptimal T cell immunogen designs.

**Methods:**

To overcome this problem, we used a novel immunogen design approach that is based on functional T cell response data from more than 1,000 HIV-1 clade B and C infected individuals and which aims to direct the T cell response to the most vulnerable sites of HIV-1.

**Results:**

Our approach identified 16 regions in Gag, Pol, Vif and Nef that were relatively conserved and predominantly targeted by individuals with reduced viral loads. These regions formed the basis of the HIVACAT T-cell Immunogen (HTI) sequence which is 529 amino acids in length, includes more than 50 optimally defined CD4^+^ and CD8^+^ T-cell epitopes restricted by a wide range of HLA class I and II molecules and covers viral sites where mutations led to a dramatic reduction in viral replicative fitness. In both, C57BL/6 mice and Indian rhesus macaques immunized with an HTI-expressing DNA plasmid (DNA.HTI) induced broad and balanced T-cell responses to several segments within Gag, Pol, and Vif. DNA.HTI induced robust CD4^+^ and CD8^+^ T cell responses that were increased by a booster vaccination using modified virus Ankara (MVA.HTI), expanding the DNA.HTI induced response to up to 3.2% IFN-γ T-cells in macaques. HTI-specific T cells showed a central and effector memory phenotype with a significant fraction of the IFN-γ^+^ CD8^+^ T cells being Granzyme B^+^ and able to degranulate (CD107a^+^).

**Conclusions:**

These data demonstrate the immunogenicity of a novel HIV-1 T cell vaccine concept that induced broadly balanced responses to vulnerable sites of HIV-1 while avoiding the induction of responses to potential decoy targets that may divert effective T-cell responses towards variable and less protective viral determinants.

**Electronic supplementary material:**

The online version of this article (doi:10.1186/s12967-015-0392-5) contains supplementary material, which is available to authorized users.

## Background

HIV-1 infection induces strong and broadly directed HLA class I and class II restricted T-cell responses, for which some specific epitopes and restricting HLA alleles have been associated with relative *in vivo* virus control or lack thereof [1-3]. Among these, CD8^+^ cytotoxic T lymphocytes (CTL) responses to HIV-1 Gag have most consistently been associated with reduced viral loads in both HIV-1 clade B- and C-infected cohorts [2,4]. This is in line with data from post-hoc analyses of the STEP vaccine trial, where individuals in whom vaccine-induced responses targeted ≥3 different Gag epitopes achieved a lower viral load than subjects without Gag responses [5]. CD4^+^ T-cell responses to Gag have also been associated with relative HIV-1 control [6,7]. However, it remains unclear whether the relative benefit of Gag is due to high protein expression levels, rapid representation of viral particle-derived CTL epitopes [8], reduced susceptibility of Gag-specific CTL to Nef-mediated immune evasion strategies [9] or particular amino acid composition and inherently greater immunogenicity [10]. In addition, the elevated level of conservation of Gag across viral isolates [11] and the severe fitness reductions caused by CTL escape variants [12-16] may provide Gag-specific T-cell responses with a particular advantage. At the same time, it is also clear that not all Gag-specific responses exert the same antiviral activity, suggesting that a rational selection of Gag components could help focus vaccine induced responses onto the most protective targets. The same likely applies for all other viral proteins as well, as they may contain some regions that are of particular value for inclusion in a vaccine while other regions or proteins may induce less useful T cell responses. As such, effective vaccine design should probably aim to induce broad and evenly distributed responses to conserved and vulnerable sites of the virus while avoiding the induction of responses to regions that can be highly immunogenic but that may act as potential “decoy” targets and divert responses away from more relevant targets [17-22].

The failure of various T-cell vaccine candidates expressing entire HIV-1 proteins in large human clinical trials and data from post-trial analyses suggesting a sieve effect on the infecting viral strains, indicate the urgent need to improve vaccine immunogen design [23-26]. Here, we describe a rational design and pre-clinical testing of a novel approach to HIV-1 T cell immunogen development and its implication for HIV-1 control. Starting with a comprehensive screening of large cohorts of clade B and C HIV-1-infected individuals, we identified viral targets associated with relative HIV-1 control [27,28]. These earlier analyses in aggregate identified 26 regions in HIV-1 Gag, Pol, Vif and Nef proteins that (i) were preferentially targeted by individuals with low viral loads and largely independent on beneficial HLA class I genotypes, (ii) turned out to be more conserved than the rest of the proteome, and (iii) elicited responses of higher functional avidity and broader variant cross-reactivity than responses to other regions. These identified regions provided the basis for a polypeptide sequence that is designed to a) contain epitope-rich regions in the context of a broad HLA class I and class II allele coverage, b) induce responses to subdominant epitopes associated with viral control and c) focus the vaccine-induced response onto the most vulnerable targets in the viral proteome. The conserved elements (CE) immunogen designs, although modified using immune response and virologic control data, was driven first by sequence conservation [17-19]. The rational design of this novel HTI sequence also differs conceptually from other approaches that have either been based on full protein sequences [23-25], on very short, conserved segments of the virus [29,30] select conserved CD8 T cell epitopes [31] or other approaches that attempt to cover viral diversity [32-35] and which did not incorporate human immune reactivity data as the base of their designs. The immune responses observed in preclinical testing in C57BL/6 mice and Indian rhesus macaques show the expected broadly directed responses and warrant further development in human clinical trials.

## Methods

### HIV-1 infected patients and human PBMC samples for ELISPOT analyses

Chronically HIV-1 infected individuals not receiving antiretroviral treatment that fulfilled the following criteria were recruited from the HIV Unit in Hospital Germans Trias i Pujol, Badalona and Hospital de la Vall d’Hebron, Barcelona, Spain: chronic HIV-1 infection with (i) persistent undetectable viral loads (Elite controllers, n = 38), (ii) stable plasma HIV-1 RNA levels <2,000 copies/ml (Viremic controllers, n = 27), and (iii) non-controlled viral replication with current plasma HIV-1 RNA levels >50,000 copies/ml (Non-controllers, n = 30) (Table 1). The study was approved by the Institutional Review Board of both participating hospitals, and all individuals provided written informed consent before entering the study. HLA genotypes were heterogeneous in all groups (data not shown). PBMC were processed within 4 h after venepuncture and cells stored at liquid nitrogen until use. A set of 410 overlapping-peptides (18-mers OLPs) was used to screen for HIV-specific T-cell responses to the full HIV-1 proteome. The peptides spanned all HIV-1 proteins and were used in a matrix layout of 6–12 peptides per pool for comprehensive screening as previously described [36]. All positive wells in the matrix screen were confirmed the following day on a single-peptide level. Results are presented as the contribution of responses targeting OLPs included in the HTI sequence relative to the total response to the full HIV-1 proteome.

**Table 1:** **Clinical data of cohort for human PBMC samples** ^**a**^ **and HLA class I allele distribution**

**Group (N)**	**HIV-1 RNA levels (copies/ml)**	**CD4** ^**+**^ **T cells (cells/mm** ^**3**^ **)**	**Protective HLA alleles**
EC (38)	UD^b^	826 (606; 946)	B*27 (8%) B*57 (26%) B*58 (3%)
VC (27)	540 (265; 1,182)	642 (498; 1,014)	B*27 (4%) B*57 (18%) B*58 (7%)
NC (30)	140,00 (58,000; 415,000)	128 (59; 211)	B*27 (7%) B*57 (7%) B*58 (7%)

^a^Values expressed as median (IQR).

^b^UD, undetectable viremia (<49 copies/ml).

### DNA.HTI and MVA.HTI vaccines

The HTI plasmid DNA (plasmid 298H) contains the expression-optimized [37-39] HTI open reading frame inserted into a pCMVkan vector comprising a plasmid backbone optimized for growth in bacteria, the human cytomegalovirus (CMV) promoter without any introns, the optimized HTI gene, the bovine growth hormone (BGH) polyadenylation site, and the kanamycin resistance gene. The HTI gene contains the human GM-CSF signal peptide (AA 1–17; Genbank accession Nr. NP_000749) at the N terminus as a means to enhance translocation into the Endoplasmatic Reticulum [40]. A FLAG-tag was added at the C–terminus (HTI-FLAG) for expression analysis in transfected HEK293 cells. RNA/codon optimized clade B (strain HXB2) genes for full-length p55^gag^ (plasmid 114H), Pol (plasmid 133H), NefTatVif fusion (NTV, plasmid 132H), and LAMP-Pol (plasmids 128H) and LAMP-NTV (plasmids 129H), fusion proteins with the lysosomal associated membrane protein 1 (LAMP-1) [40] were used. The IL-12 DNA (plasmid AG157) produces the expression-optimized rhesus macaque IL-12 cytokine [41]. Plasmid DNAs were produced in *E.coli* DH10B (Invitrogen), and the purified endotoxin-free DNAs (Qiagen) were resuspended in sterile water (Gibco).

A recombinant MVA expressing the HTI gene was generated as described previously [42,43]. Briefly, chicken embryo fibroblast (CEF) cells grown in Dulbeco’s Modified Eagle’s Medium supplemented with 10% FBS, penicillin/streptomycin and glutamine (DMEM 10) were infected with parental MVA and transfected using Superfectin (Qiagen) with DNA.HTI. These cells were MoFlo single-cell sorted into 96-well plates and these were used to culture recombinant virus upon addition of fresh CEF. Those wells containing suitably infected cells were harvested and screened by PCR to confirm identity and test purity. Plaque picking was performed using CEF until the culture was free of parental virus, as determined by PCR, after which a master virus stock was grown, purified on a 36% sucrose cushion, titered and stored at −80°C until use.

### HTI DNA expression upon transient transfection

Briefly, 1 × 10^6^ human HEK 293 cells grown in complete DMEM plus 10% fetal bovine serum (FBS) were plated on to 60-mm tissue culture dishes and allowed to adhere overnight. Cells were transfected by CaPhosphate DNA co-precipitation with a total of 7 μg of DNA (100 ng or 250 ng of the plasmid DNA adjusted to 7 μg with Bluescript DNA). Six hours after transfection the medium was replaced with 3 ml of DMEM supplemented with 2% of FBS. After 24 and 48 hrs the cells and the supernatants were collected in 0.5X RIPA (Boston BioProducts, Ashland, MA). Protein expression was analyzed by Western immunoblots using 10% or 12% sodium dodecyl sulfate polyacrylamide gels (Nu-Page Bis-Tris, NuPAGE, Invitrogen, Life Technologies Corp., Carlsbad, CA) and blotted onto nitrocellulose membranes which were probed with a anti-FLAG-HRP antibody (dilution 1:4000, Sigma) or a goat anti-p24^gag^ antibody (dilution 1:3000, provided by L. Arthur, SAIC, NCI, Frederick) followed by anti-goat IgG-HRP labeled antibody (dilution 1:10,000; Calbiochem, EMD chemicals, Gibbstown, NJ). As control, the membranes were probed with anti-human pan-actin antibody (clone C4, EMD Millipore, Billerica, MA) at a dilution of 1:10,000. The bands were visualized using the enhanced chemiluminescence (ECL) plus Western blotting detection system (GE HealthCare, Piscataway, NJ). HeLa-derived HLtat cells were plated with 2 × 10^5^ cells/35-mm glass-bottomed plate and 24 hrs later were transfected with 500 ng of the DNA.HTI-FLAG plasmid. The next day, the cells were fixed with 4% paraformaldehyde in PBS, permeabilized with 0.5% Triton X-100 in PBS and incubated with mouse anti-FLAG antibody (Sigma) at 1:1,000 dilution, followed by incubation with anti-mouse Alexa-fluor 488 and DAPI at 1:1000 dilution. The cells were visualized on a Zeiss Observer Z1 fluorescent microscope using Zeiss Axiovision software (Carl Zeiss Microimaging, GmbH, Göttingen, Germany).

### Vaccination of C57BL/6 mice

Groups of five female C57BL/6 (6 to 8 weeks old) were obtained from Charles River Laboratories, Inc. (Frederick, MD) and were housed at the National Cancer Institute, Frederick, MD, in a temperature-controlled, light-cycled facility. The mice were immunized with DNA.HTI or DNA.COMB consisting of *gag*, *pol* and NTV (*nef, tat, vif*) expression plasmids using doses of 20 μg DNA per mouse. The DNA was delivered by intramuscular injection followed by *in vivo* electroporation by ELGEN® constant current electroporation device (Inovio Pharmaceuticals, Inc, Blue Bell, PA). DNA.COMB consists of DNA.Gag clade B (expressing complete clade B p55 Gag protein) in combination with DNA.Pol (expresses full-length Pol protein) and DNA.NTV (expressing a chimeric protein of Nef, Tat and Vif) plasmids. The animals were vaccinated twice (0 and 3 weeks or 0 and 4 weeks), and were sacrificed 2 weeks after the last vaccination when spleens and blood were collected for the analysis of cellular and humoral responses.

For heterologous prime/boost experiments, groups of 6 C57BL/6 mice (Harlan Laboratories Ltd., Barcelona, Spain) were used in the Hospital Germans Trias i Pujol Animal facility. Mice were primed intramuscularly with 100 μg of DNA.HTI (2, 3 or 4 vaccinations) followed by a 10^6^ pfu of MVA.HTI boost, as indicated. All vaccinations were separated by three weeks. All mice were sacrificed two weeks after the last vaccination when splenocytes and serum were harvested for immunogenicity studies. Spleens were removed and pressed individually through a cell strainer (Falcon) using a 5-ml syringe rubber plunger. Following red blood cell lysis, splenocytes were washed and resuspended in RPMI 1640 supplemented with 10% FCS, penicillin/streptomycin (R10) and frozen until use.

### Overlapping peptides and distribution of peptide pools

To allow for comparisons in immunogenicity among groups of animals, sequences of the immunogenic segments contained in HTI were confirmed to be >95% matched with the sequences from the full-length proteins expressed by control DNAs. *Ex vivo* immune analyses of murine experiments in which DNA expressing full proteins were used, employed a previously described overlapping peptide set (410 18-mers OLP) spanning the entire viral proteome [36]. Peptides were 18-mers varying from 15–20 amino acids in length and overlapping by 10 amino acids, designed using the PeptGen algorithm at the Los Alamos HIV database [44] and based on the 2001 consensus-B sequence [2,36]. To assess responses to entire Gag, Pol, Nef, Tat and Vif and the relative distribution of responses to regions covered by HTI T-cell immunogen, a set of peptide pools was designed that consisted of 6 pools for Gag (11 peptides/each), 8 for Pol (16 or 17 peptides/each), 2 for Nef (13 and 14 peptides/each), 1 for Tat (12 peptides) and 2 for Vif (12 peptides/each) proteins. A second peptide set consisting of 8 peptide pools covered the protein subunits spanning the 16 segments included in the HTI and was used in the experiments where electroporation was performed. For experiments that assessed the immune responses after heterologous prime/boost vaccinations and for analyses that measured the potential immunogenicity of junctional epitopes an overlapping peptide set of 147 peptides of 15 amino acids in length (overlapping by 11 residues) spanning the entire HTI (including the leader sequence and linkers regions) was synthesized. For mice experiments, HTI peptides were pooled into 18 different pools, according to protein subunits and considering a similar number of peptides per pool (1 pool for the signal peptide sequence, n = 4 peptides; 7 pools for Gag, n = 8-11 peptides/each; 7 pools for Pol, n = 5-11 peptides/each; 2 pools for Vif, n = 6-8 peptides/each and 1 pool for Nef, n = 2 peptides). In macaque experiments and to assess breadth of vaccine induced responses, peptides were pooled and distributed covering each HTI segment individually (S1-S16).

### Murine IFN-γ ELISPOT assay

ELISPOT assay was performed by using a mouse IFN-γ ELISPOT kit (ALP) (Mabtech AB, Stockholm, SE) following the manufacturer’s instructions with minor modifications. For all assays, frozen splenocytes were thawed and rested for 5 hrs at 37°C in R10 medium before use. Cells were added at an input cell number of 4 × 10^5^ cells/well in 140 μl of R10 in 96-well polyvinylidene plates (Millipore Corp., Bedford, MA) alone or with HIV-1- specific peptide pools (14 μg/ml final concentration for each peptide) for 16 hrs at 37°C in 5% CO_2_. Concanavalin A (Sigma-Aldrich Corp., Saint Louis, MO), at 5 mg/ml, was used as a positive control. The plates were developed with one-step 5-bromo-4-chloro-3-indolyl phosphate/Nitroblue Tetrazolium (BCIP/NBT, Bio-Rad Laboratories, Inc., Irvine, CA). The spots on the plates were counted using an automated ELISPOT reader system (CTL Analyzers LLC, Cleveland, OH) using ImmunoSpot software and the magnitude of responses was expressed as spot forming cells (SFC) per million splenocytes. The threshold for positive responses was defined as at least 5 spots per well and responses exceeding the mean number of spots in negative control wells plus 3 standard deviations of the negative control wells and three times the mean of negative control wells, whichever was higher.

### Humoral immune response analysis

Humoral responses were analyzed in pooled mice plasma. Binding antibodies to HIV-1 Gag were detected by Western immunoblot using cell extracts from HEK293 cells transfected with 1 μg of Gag and Gag-Pol expression vectors, separated on 12% SDS-PAGE, and the membranes were probed with pooled plasma (at a 1:100 dilution) and the bands were visualized by anti-mouse IgG-HRP antibody (1:10,000 dilution, GE Healthcare, Piscataway, NJ). Serial dilutions of plasma samples were analyzed by standard HIV-1 clade B p24^gag^ ELISA (Advanced Bioscience Lab, Rockville, MD), measuring optical absorbance at 450 nm.

### Vaccination of rhesus macaques

Indian rhesus macaques (R678, R679, R680, R681) were vaccinated with 2 mg of DNA.HTI together with 0.2 μg of macaque IL-12 DNA formulated in 0.6 ml of sterile water (Hospira, Inc., Lake Forest, IL). DNAs were delivered via intramuscular (IM) injection at two different sites (0.3 ml each site) followed by *in vivo* electroporation using the Elgen 1000 device (Inovio, Pharmaceuticals, Inc, Blue Bell, PA). The animals received three vaccinations with DNA.HTI at 0, 1 and 3 month. After a rest period of 4.5 months, the animals received a boost with recombinant MVA.HTI (10^8^ pfu/dose) delivered via the IM route as a single inoculation. A 2nd MVA.HTI boost was administered 3 months later. All macaques used in this study were males and were 3 years of age at the onset of the study. Vaccinations were performed under anesthesia (Ketamine administered at 10 mg/kg) and all efforts were made to minimize suffering. No adverse effects were found. None of the animals were euthanized as part of this study.

### Intracellular cytokine staining of macaque PBMC

Macaque PBMC were isolated by Ficoll-hypaque (Histopaque, Sigma, St. Louis, MO) centrifugation, and cultured in 96-well plates as described previously [45]. PBMC were stimulated overnight with peptide pools (final concentration of 1 μg/ml for each peptide) in the presence of monensin (BD Pharmingen, San Diego, CA), Antigen-specific T cells were monitored by intracellular cytokine staining followed by polychromatic flow cytometry. The cells were stained with the following cocktail of cell surface antibodies: CD3-APCCy7 (clone SP34-2), CD4-V500 (clone L200), CD95-FITC (clone DX2) (BD Pharmingen), CD8-Alexa Fluor-405 (clone 3B5, Invitrogen, Carlsbad, CA), CD28-PerCP Cy5.5 (clone CD28.2, BioLegend, San Diego, CA). CD107a-eFluor 660 (clone eBioH4A3, eBioscience San Diego, CA) antibody was added to the cells 10 min after addition of the peptides. After cell permeabilization with Cytofix/Cytoperm (BD Biosciences), intracellular staining was performed using IFN-γ-PE Cy7 (clone B27, BD Pharmingen) and Granzyme B-PE antibodies (clone GB12, Invitrogen). In all experiments, PBMC cultured in medium without peptide pools or stimulated with phorbol myristate acetate (PMA) and calcium ionophore (Sigma, St. Louis, MO) were used as negative and positive control, respectively. Samples were considered positive, if the frequency of the IFN-γ^+^ T cells of the peptide stimulated sample was more than 2 fold higher than the frequency obtained in unstimulated (without peptide) medium only control sample. At least 10^5^ T cells from each sample were acquired on an LSR II flow cytometer (BD Biosciences, San Jose, CA) and the data were analyzed using FlowJo software (Tree Star, Inc., Ashland, OR). Subsets of antigen-specific T cells were defined by Boolean gating, including central memory (CD95^+^ CD28^+^), effector memory (CD95^+^ CD28^−^) and cytotoxic potential (granzyme B and CD107a expression).

### Humoral immune responses of vaccinated macaques

HEK293 cells were transfected with 0.5 μg of HTI-FLAG expression vector and immunoprecipitated using anti-Flag M2 affinity gel (Sigma, Catalog Number A2220) to concentrate the protein 20-fold. The proteins were separated on 12% SDS-PAGE and transferred to membrane as described above. The membranes were probed with plasma from the vaccinated macaques at a 1:100 dilution, and the bands were visualized using a 1:10,000 dilution of an anti-monkey IgG+, IgA+, IgM + −HRP-conjugated antibody (cat# 43R-IG050hrp; Fitzgerald Industries International Inc., MA). The bands were visualized using the enhanced chemiluminescence (ECL) plus Western blotting detection system (GE HealthCare, Piscataway, NJ). ELISA was performed using serial 4-fold dilutions of plasma samples analyzed by standard HIV-1 clade B p24^gag^ ELISA (Advanced Bioscience Laboratory, Rockville, MD), measuring optical absorbance at 450 nm.

### Statistical analyses

The results are given as medians and interquartile range as indicated unless otherwise stated. GraphPad Prism version 4.0 for Windows (San Diego, CA) was used to compare response rates in both groups and subgroup analyses. Mann–Whitney test and Wilcoxon matched paired test were used for unpaired and paired comparisons, respectively.

### Ethics statement

Chronically HIV-1 infected adult individuals were recruited from the HIV Unit in Hospital Germans Trias i Pujol, Badalona and Hospital de la Vall d’Hebron, Barcelona, Spain through the prospective observational cohort study named ‘Controllers’. The study was approved by the Institutional Review Board of Hospital Germans Trias i Pujol, Badalona and Hospital de la Vall d’Hebron, Barcelona. All adult subjects provided written informed consent before entering the study.

C57BL/6 mice used for this study were cared at the temperature-controlled, light-cycled animal facilities of National Cancer Institute, Frederick, US and Hospital Germans Trias i Pujol, Badalona, Spain. The animal user protocol was approved by the NCI-Frederick Animal Care and Use Committee (AWA#:A4159-01). Frederick National Laboratory for Cancer Research is accredited by AAALAC International and follows the Public Health Service Policy for the Care and Use of Laboratory Animals. Procedures involving mice performed at the animal facility of Hospital Germans Trias i Pujol followed EU normative on animal experimentation and were approved by the Animal Experimentation Ethical Committee from the Institut d’Investigació Sanitària Germans Trias i Pujol and the Department d’Agricultura, Ramaderia, Alimentació i Medi Natural of the Generalitat de Catalunya (DAAM order number 6390).

Indian rhesus macaques (Macaca mulatta) used in this study were housed at the Bioqual (formerly Advanced BioScience Laboratories, Inc.) animal facility. All animals were cared for and procedures performed under a protocol approved by the ABL Animal Care and Use Committee (animal welfare assurance no. A3467-01; protocol no. AUP572) and USDA Certificate number 51-R-0059. The macaques were managed according to the animal husbandry program, which aims at providing consistent and excellent care to nonhuman primates at the vivarium. This program operates based on the laws, regulations, and guidelines promulgated by the United States Department of Agriculture (e.g., the Animal Welfare Act and its regulations, and the Animal Care Policy Manual), Institute for Laboratory Animal Research (e.g., Guide for the Care and Use of Laboratory Animals, 8th edition), Public Health Service, National Research Council, Centers for Disease Control, and the Association for Assessment and Accreditation of Laboratory Animal Care (AAALAC) International. The nutritional plan utilized by the Animal Facility consisted of twice daily feeding of Labdiet 5045 High Protein Primate Diet and food intake was closely monitored by animal research technicians. This diet was also supplemented with a variety of fruits, vegetables, and other edible objects as part of the environmental enrichment program established by the Veterinary staff and enrichment Technician. Pairing of animals as part of the environmental enrichment program was managed by the enrichment technician. All primary enclosures and animal rooms were cleaned daily with water and sanitized at least once every two weeks. All macaques (N = 4) used in this study were males and were 3 years of age at the onset of the study. Vaccinations were performed under anesthesia (Ketamine administered at 10 mg/kg) and all efforts were made to minimize suffering. No adverse effects were found. None of the animals were euthanized as part of this study.

## Results and discussion

### Design of the HIVACAT T-cell immunogen (HTI) covering targets of effective antiviral T cell responses

We recently reported a systematic analysis of a large set of human immune data from cohorts of HIV-1 infected individuals [27,28,36]. In these analyses, more than 1,000 untreated HIV-1 clade B and clade C infected individuals were screened for anti-HIV T cell responses and regions of the viral proteome predominantly targeted by subjects with superior HIV-1 control were revealed. Of the 410 18-mer overlapping peptides (OLP) spanning the entire viral proteome, 26 consensus clade B derived OLP were identified for which the median viral load in the OLP responder group was significantly lower than the viral load in subjects who did not target these OLP (i.e., OLP non-responders). These beneficial OLP were located in the HIV-1 Gag (n = 10), Pol (n = 12), Vif (n = 3) and Nef (n = 1) proteins (Table 2) and overlapped or closely matched the beneficial regions identified by analogous screening in HIV-1 clade C-infected subjects [28].

**Table 2:** **List of identified beneficial overlapping peptides (OLP, PR > 1) incorporated in the final T-cell immunogen design, based on previous analysis** [28]

**OLP no.**	**Protein**	**Subunit**	**OLP clade B cons sequence**
3	Gag	p17	EKIRLRPGGKKKYKLKHI
6	Gag	p17	ASRELERFAVNPGLL
7	Gag	p17	ERFAVNPGLLETSEGCR
10	Gag	p17	QLQPSLQTGSEELRSLY
12	Gag	p17	SLYNTVATLYCVHQRIEV
23	Gag	p24	AFSPEVIPMFSALSEGA
31	Gag	p24	IAPGQMREPRGSDIA
34	Gag	p24	STLQEQIGWMTNNPPIPV
48	Gag	p24	ACQGVGGPGHKARVLAEA
60	Gag	p15	GKIWPSHKGRPGNFLQSR
75	Nef	-	WLEAQEEEEVGFPVRPQV
159	Pol	Prt	KMIGGIGGFIKVRQYDQI
160	Pol	Prt	FIKVRQYDQILIEICGHK
161	Pol	Prt	QILIEICGHKAIGTVLV
163	Pol	Prt	LVGPTPVNIIGRNLLTQI
171	Pol	RT	LVEICTEMEKEGKISKI
195	Pol	RT	LRWGFTTPDKKHQKEPPF
196	Pol	RT	DKKHQKEPPFLWMGYELH
210	Pol	RT	EIQKQGQGQWTYQIY
269	Pol	Int	TKELQKQITKIQNFRVYY
270	Pol	Int	TKIQNFRVYYRDSRDPLW
271	Pol	Int	YYRDSRDPLWKGPAKLLW
276	Pol	Int	KIIRDYGKQMAGDDCVA
405	Vif	-	VKHHMYISGKAKGWFYRH
406	Vif	-	GKAKGWFYRHHYESTHPR
424	Vif	-	TKLTEDRWNKPQKTKGHR

The 26 beneficial OLP were aligned and if located in close proximity (<4 amino acid residues between one end and the start of an adjacent beneficial OLP) fused using the naturally occurring clade B consensus sequence residues. This resulted in 16 continuous segments with the precise start and end positions defined after considering additional residues up- and down-stream of the identified 26 OLP for inclusion. These extensions and truncations reflected: (i) extension of the segments beyond the OLP border so that additional specific epitopes that were considered important or can be presented as extended length variants were fully covered [2,3,27,46] (ii) shortening of segments to exclude immunodominant epitopes for which no published evidence supports their beneficial effects on viral control, (iii) extensions to cover flanking regions known to affect epitope processing [47,48] and (iv) introduction of single amino acid changes to cover common drug-resistance mutations for which maintained immunogenicity of the resistance variants had been reported [48-50]. Further refinement also included cases where the reported epitopes were originally not identified in the context of consensus clade B sequence [51] and sequence truncations to avoid the presence of “forbidden” residues that are essentially never found on C-terminal ends of HLA class I restricted T-cell epitopes [44,52]. Finally, two additional regions in Gag were incorporated to complete the coverage of previously described hot-spots of CD4^+^ T-helper cell activity [53]. These two segments partially overlapped with two of the most conserved regions of Gag p24 [19] that had been shown to be preferentially targeted by HIV-1 controllers and also include the protective B27 KK10 and B14 DA9 CTL epitopes [27]. The final sequence comprised 16 segments ranging from 11–78 amino acids in length representing Gag (45%), Pol (44%), Vif (8%) and Nef (3%) sequences (Table 3). Linkers between segments consisted of either single, dual or triple alanine residues, and were included with the aim of inducing preferential proteolytic cleavage between segments and to avoid premature epitope digestion [47,54].

**Table 3:** **Distribution and length of the 16 final HIV-1 segments included in the HTI sequence**

**HTI segments**	**Length (AA)**	**HIV-1 protein**
S1	78	Gag-p17
S2	14	Gag-p24
S3	11	Gag-p24
S4	60	Gag-p24
S5	14	Gag-p24
S6	15	Gag-p24
S7	27	Gag-p15
S8	55	Prt
S9	17	RT
S10	55	RT
S11	34	RT
S12	34	Int
S13	17	Int
S14	26	Vif
S15	19	Vif
S16	13	Nef
Total (incl. linker)	529	

**Figure 1:**
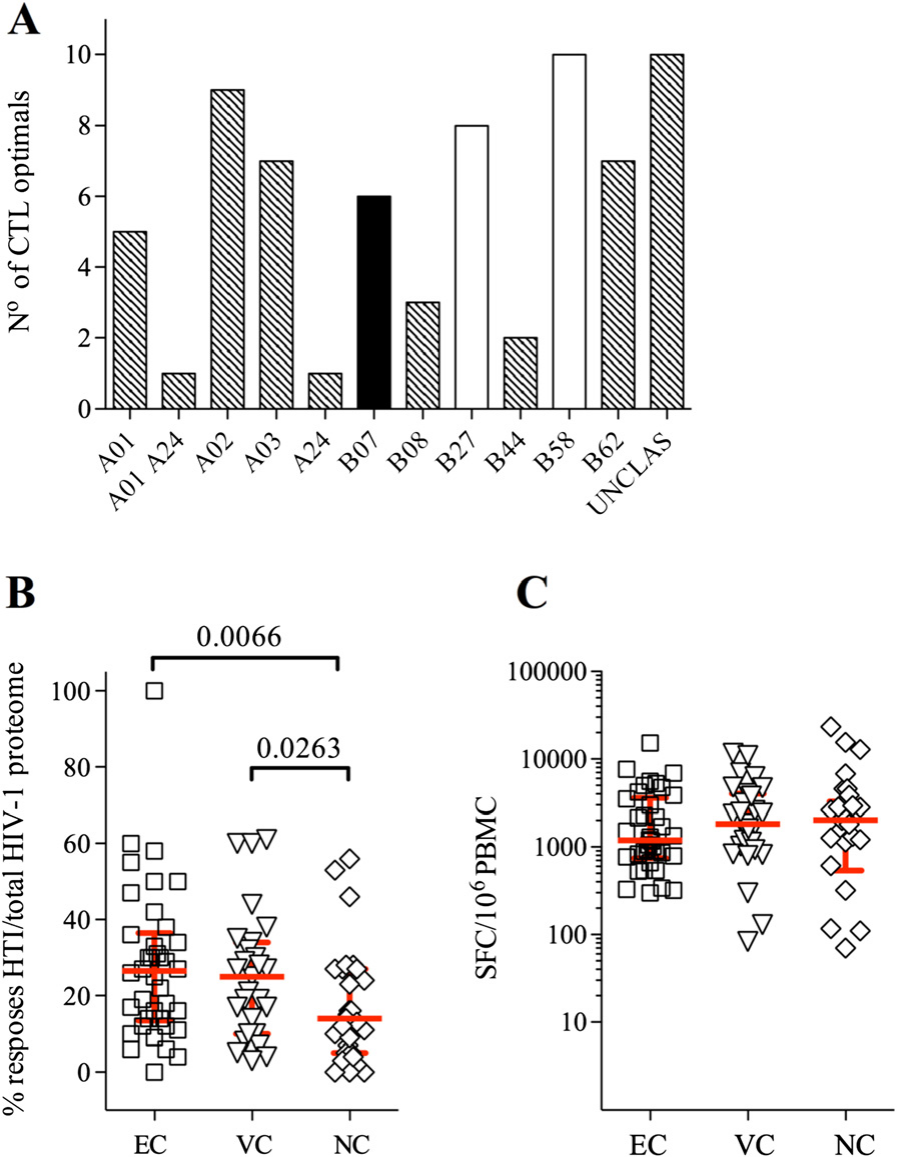
**Design of HTI, HLA coverage and translation into HIV-1 control. (A)** HLA supertype coverage of all the optimal defined epitopes included in HTI immunogen [52]. Colors are based on the allele’s reported relative hazard to develop AIDS (grey, neutral; black, neutral/rapid progression; white, beneficial) [57,58]. **(B)** The relative dominance of the responses induced by OLP covering the HTI sequence (n = 52) to the complete HIV-1 proteome (n = 410) is shown in elite controllers (EC, n = 38), viremic controllers (VC, n = 27) and non-controllers (NC, n = 30). **(C)** Total IFN-γ ELISPOT responses (magnitude) against the complete HIV-1 proteome elicited by consensus B overlapping 18-mer peptide sets (n = 410).

The final linear HTI sequence had a total length of 529 amino acids and included a high density of both CD8^+^ and CD4^+^ T-cell epitopes across all protein subunits restricted by at least 42 different HLA alleles (n = 55 well-characterized optimal defined CTL epitopes [55] and 6 most frequently targeted CD4^+^ T helper epitopes in Gag [53], Table 4). Of note, 14 Gag CD4^+^ T cell ‘promiscuous’ epitopes (recognized in the context of two or more DRB alleles) [7] were covered by the final HTI sequence as well. Importantly, no overrepresentation of HLA-B27 or HLA-B57 supertype-restricted epitopes was observed, which is in line with the unbiased general population screening that provided the data for the identification of beneficial OLP signal (Figure 1A). The sequence was also analyzed for its coverage of particularly vulnerable immune targets based on recently published viral fitness data for Gag p24 sequence mutations [56]. Of the 135 Gag p24 sites analyzed by Rihn and colleagues [56], 72 were covered by HTI. Of these, 58 (80%) showed high vulnerability for mutations and caused massive (>50 fold) reductions in viral replicative fitness. This was a significantly higher proportion of vulnerable sites than what was observed for the Gag p24 region that was not covered by HTI (a total of 37 deleterious mutants among 63 tested variants, p = 0.005), demonstrating further the preferential coverage of vulnerable sites by HTI.

**Table 4:** **CTL optimal epitopes and CD4** ^**+**^ **T-helper epitope coverage of HTI**

**Protein location**	**# of CTL epitopes** ^**a**^	**# of CD4** ^**+**^ **T Helper Gag epitopes (>25% targeted)** ^**b**^	**# of ‘promiscuous’ CD4** ^**+**^ **T cell Gag peptides** ^**c**^
Gag-p17	17	3	6
Gag-p24	21	2	6
Gag-p15	3	1	2
Pol-Prt	5		
Pol-RT	6		
Pol-Int	1		
Vif	2		
Nef	0		
Total	55	6	14

^a^based on the ‘A’ list of optimal defined HIV-1 CTL epitopes described in the Los Alamos HIV Immunology Database [55].

^b,c^described CD4 T-helper epitopes [7,53].

To confirm that the final HTI sequence design (i.e., including extensions, truncations and substitutions) contained T cell targets preferentially recognized by individuals with reduced viral loads, an independent cohort of HIV-1 clade B infected individuals was tested for responses to the HTI sequence and compared to responses to the full HIV-1 proteome. Data from 38 elite HIV controllers (EC), 27 viremic controllers (VC) and 30 non-controllers (NC) was collected (Table 1). A significantly stronger focus of the total virus-specific response on targets covered by the HTI was observed in both elite controllers (26.5% of total HIV-1 T-cell reactivity targeting HTI) and viremic controllers (25%) compared to individuals with uncontrolled HIV-1 infection (14%, p = 0.0066 and p = 0.0263, respectively) (Figure 1B). These differences were not due to the presence of stronger responses in HIV controllers, as the total magnitude of responses did not differ between groups (Figure 1C). Together, these data indicate that the rational design of HTI resulted in a relatively short, yet highly immunogenic subunit sequence that was especially reactive in HIV controllers and covered a particularly high proportion of vulnerable viral sites.

### Expression of HTI plasmid DNA from HEK293 cells

**Figure 2:**
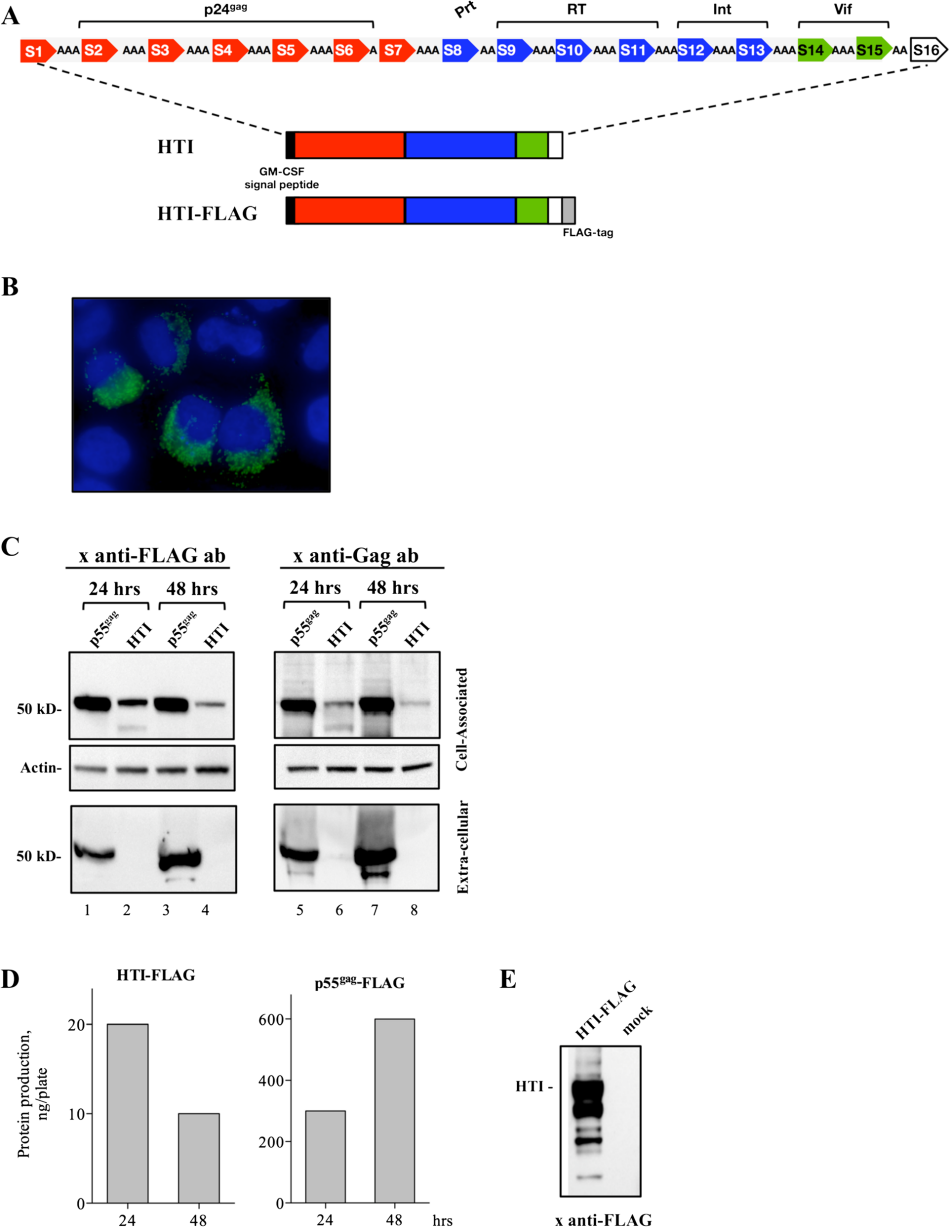
**Expression of DNA.HTI. (A)** The HTI protein is composed of 16 individual segments arranged linearly and linked via 1–3 alanine amino acid linkers and contains the GM-CSF signal peptide for better secretion. Total length of HTI protein is 529 aa, including alanine linkers. **(B)** Subcellular localization of HTI protein is shown in HeLa-derived HLtat cells transfected with DNA.HTI-FLAG and fixed. The HTI-FLAG protein was visualized with anti-FLAG primary antibody followed by Alexa-Fluor 488 conjugated secondary antibody and the nuclei were visualized with DAPI and a merged image is shown. **(C)** Plasmid DNA (250 ng) expressing HTI or p55^gag^ with or without FLAG were transfected in HEK293 cells. The cultures were harvested after 24 hrs and 48 hrs and proteins in cell-associated (top panel: 1/100) and extra-cellular (bottom panel: 1/150) fractions were resolved on a 12% NuPAGE Bis-Tris gel. Western immunoblots were analyzed using an anti-FLAG-HRP antibody (lanes 1–4) or a goat anti-p24^gag^ antiserum followed by anti-goat IgG-HRP labeled antibody (lanes 5–8) and were visualized using enhanced ECL. The membranes were probed with an anti-beta actin antibody to control for equal loading of the cell-associated fractions. **(D)** The serially diluted extracts with cell-associated and extra-cellular fractions for p55^gag^-FLAG and cell-associated fraction of HTI-FLAG proteins were analyzed together with FLAG-tagged p55^gag^ protein standard. The HTI-FLAG and p55^gag^ proteins were quantified by fitting standard curve using standard p55^gag^-FLAG protein. **(E)** Analysis of a 20-fold concentrated sample (compared to panel C) containing HTI-FLAG by Western immunoblot using anti-FLAG antibody.

The HTI sequence was RNA-codon optimized to maximize mRNA processing, transport, stability and translation and was cloned into the pCMVkan expression vector [59] (DNA.HTI). To promote production and secretion of the immunogen, the signal peptide from the human granulocyte macrophage colony-stimulating factor (GM-CSF) was added at the amino-terminus of HTI (Figure 2A) [17,18,40]. Upon transient transfection, we analyzed expression of the FLAG-tagged HTI by fluorescent microscopy and found that the protein localized to punctate foci in the cytoplasm of HeLa cells (Figure 2B). The localization of HTI protein was also evaluated from cell extracts and supernatants of transiently transfected HEK293 cells by Western immunoblots (Figure 2C) probed with an anti-FLAG (lanes 1–4) or an anti-p24^gag^ antibody (lanes 5–8). The HTI protein migrated at ~50 kDa and was only found in the cell-associated fraction (Figure 2C). We noted lower levels of HTI protein at 48 hrs compared to 24 hrs post transfection (lanes 2 and 4; lanes 6 and 8). To quantitate production from transfected cells, serial dilutions of the p55^gag^-FLAG and HTI-FLAG proteins were analyzed by Western blots using the common FLAG epitope and a standard FLAG-tagged p55^gag^ protein. A side-by-side comparison in transfected HEK293 cells showed that expression of p55^gag^ increased ~2x over time (~280 ng at 24 hrs; ~600 ng at 48 hrs), while HTI levels were ~2x decreased (~22 ng at 24 hrs; ~9.5 ng at 48 hrs) (Figure 2D). Thus, these data indicate that HTI protein accumulates intracellularly and is significantly less stable than p55^gag^. This was further confirmed by the analysis of HTI-FLAG protein pulled down with anti-FLAG antibody, which allowed the amount of protein loaded per lane to be increased. The Western immunoblot analysis revealed several shorter bands in addition to the full-length HTI protein, possibly reflecting processed products (Figure 2E).

### DNA plasmid vaccination in C57BL/6 mice elicits broad and balanced T-cell responses to all protein components of HTI

**Figure 3:**
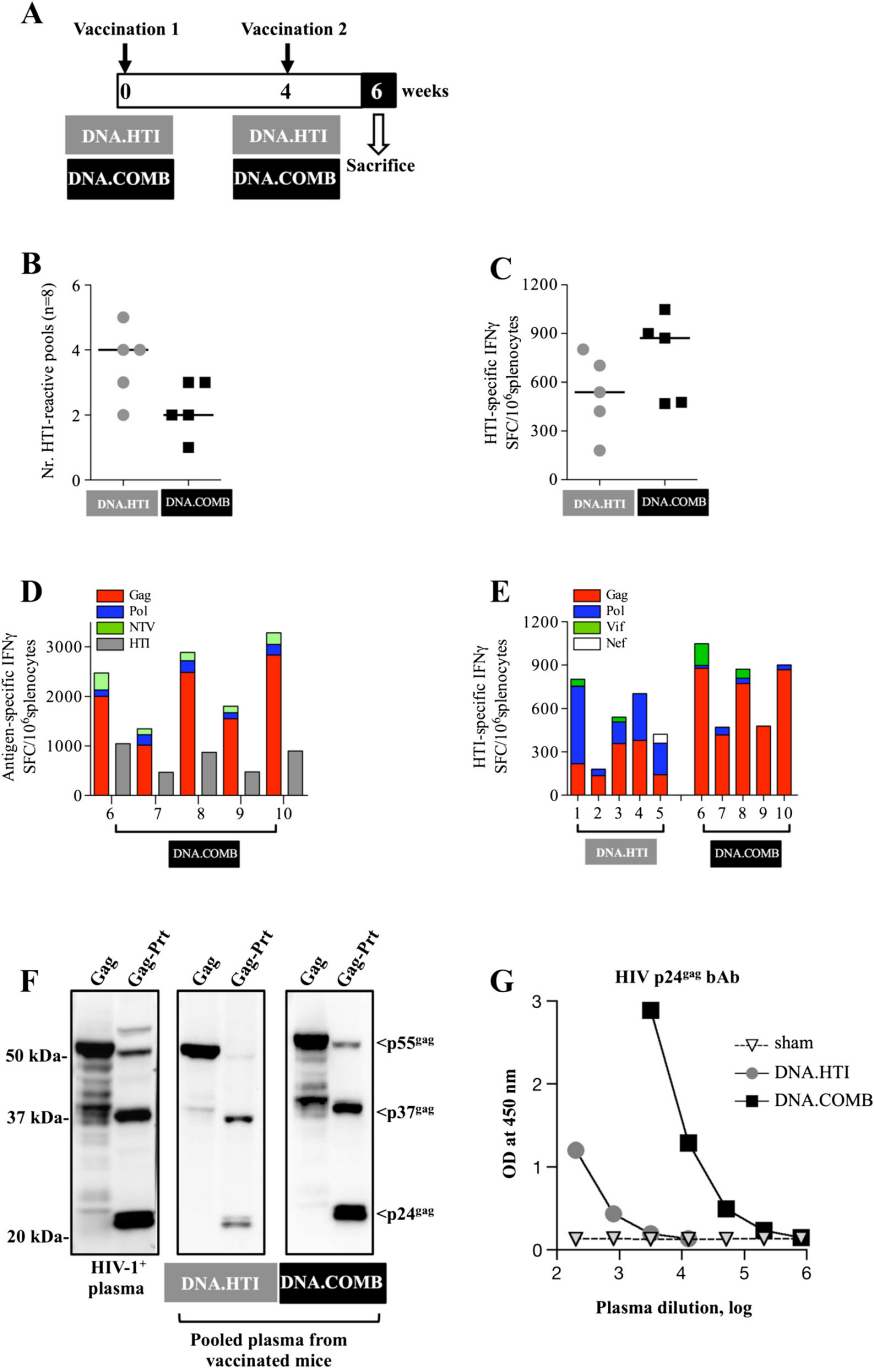
**Balanced immunogenicity upon vaccination with DNA.HTI in mice. (A)** Vaccination schedule. Groups of mice (n = 5) were vaccinated twice by IM injection followed by *in vivo* electroporation (EP) using 20 μg DNA.HTI and a mixture of 3 plasmids encoding for full-length p55^gag^, Pol and a Nef-Tat-Vif fusion protein (DNA.COMB). Cellular immune responses were measured from thawed splenocytes by IFN-γ ELISPOT 2 weeks after the 2^nd^ vaccination using 8 peptide pools covering the HTI sequence. **(B)** The number of HTI positive responses (reactive pools) in mice immunized with 20 μg DNA.HTI or DNA-COMB is shown. **(C)** Total magnitude of the HTI-specific IFN-γ responses is depicted from the mice shown in panel B. **(D)** Comparison of the IFN-γ responses in mice vaccinated with DNA.COMB targeting the regions included in the HTI (grey bars) and the total IFN-γ specific response to peptide pools spanning the complete Gag, Pol, NTV. **(E)** Distribution of HTI-specific induced IFN-γ responses against Gag, Pol, Vif and Nef in mice from panel B is shown (DNA.HTI in mice 1–5; DNA.COMB in mice 6–10) using peptide pools spanning the protein sequences included in HTI. **(F)** Binding antibodies to Gag were detected by Western immunoblot. The membranes contain Gag proteins from HEK293 cells transfected with 1 μg of p55^gag^ plasmid producing the unprocessed p55^gag^ protein or transfected with a Gag-Prt plasmid expressing p55^gag^, the processing intermediate p37^gag^ (p24^gag^ and p17^gag^) and processed p24^gag^ proteins. Membranes were probed with human sera from an HIV-infected individual or pooled plasma from mice immunized with 20 μg of DNA.HTI and DNA.COMB (at a 1:100 dilution). **(G)** Anti-HIV-1 p24^gag^ antibodies were measured in pooled plasma from DNA.HTI and DNA.COMB vaccinated C57BL/6 mice by a standard clade B p24^gag^ ELISA. The graph shows absorbance (optical density, OD).

To examine the immunogenicity of HTI, groups of five C57BL/6 mice were vaccinated twice (0 and 4 weeks) by intramuscular (IM) injection combined with *in vivo* electroporation (EP) (Figure 3A). DNA.HTI vaccination was compared to a control group that received a mixture of 3 plasmids encoding full-length p55^gag^, Pol and a Nef-Tat-Vif fusion protein (DNA.COMB). The magnitude and breadth of the vaccine-induced T-cell response were assessed in splenocytes collected 2 weeks after the 2^nd^ vaccination (week 6) by an IFN-γ ELISPOT assay using two sets of peptide pools: (i) 8 overlapping peptide pools covering the HTI sequence and assembled in separate pools for each protein subunit included in HTI (HTI pool-1 to pool-8) and (ii) 19 additional peptide pools spanning the full-length sequences of Gag (n = 6 pools), Pol (n = 8), Nef (n = 2), Tat (n = 1), and Vif (n = 2). These pools were used to assess responses to HTI itself and to define the responses directed to the HTI portion in animals vaccinated with the full-length protein sequences.

Although the HTI sequence was only a fraction of the combined length of the full-protein sequence in the DNA.COMB (529 amino acids vs 2,000 residues), equally broad responses to the 8 peptide pools covering the HTI sequence were detected after vaccination with the DNA.HTI vaccine [median of 4 reactive pools (range 2–5)] or DNA.COMB [median 2 reactive pools (range 1–3), p > 0.05] (Figure 3B). Similarly, the magnitudes of the HTI-specific responses were comparable in the two groups (Figure 3C). Importantly, six of the eight protein subunits in the HTI immunogen sequence were targeted at least once in the mice immunized with DNA.HTI, while only 4 peptide pools were reactive in animals vaccinated with DNA.COMB (p > 0.05, Table 5). In line with this data and as hypothesized, mice immunized with plasmids encoding the full-length protein sequences mounted responses largely (74%) towards regions not covered by the HTI and showed a strong dominance of Gag-specific T-cell activity (Figure 3D). Polychromatic flow cytometry analysis further revealed that the HTI-specific responses in the DNA.HTI were mediated primarily by CD8^+^ T cell (not shown). Although Gag was the most potent target in the DNA.HTI vaccinated animals as well (median of 55% of the total magnitude), the pattern of responses was shifted to a more balanced hierarchy (Gag > Pol > Vif > Nef) (Figure 3E), which was representative of the relative length of the different protein subunits in the HTI immunogen.

**Table 5:** **Distribution of HTI-specific responses and number of mice with responses to each protein subunit**

			**Number of animals with positive response (n = 5)**	
**HTI segments**	**HIV-1 protein**	**HTI pool (#peptides/pool)**	**DNA.COMB**	**DNA.HTI**
S1^a^	Gag-p17	Pool1 (10)	0	2
S2	Gag-p24	Pool2 (12)	5	5
S3	Gag-p24			
S4	Gag-p24			
S5	Gag-p24			
S6	Gag-p24			
S7	Gag-p15	Pool3 (3)	0	0
S8^a^	Prt	Pool4 (6)	2	5
S9^a^	RT	Pool5 (11)	2	4
S10^a^	RT			
S11	RT			
S12	Int	Pool6 (4)	0	0
S13	Int			
S14	Vif	Pool7 (4)	2	2
S15	Vif			
S16^a^	Nef	Pool8 (2)	0	1

^a^segments with increase in HTI-specific responses in DNA.HTI and DNA.COMB.

To determine the immunogenicity of potential junctional neo-epitopes located between the 16 segments of the HTI sequence, all individual overlapping peptides that contained at least one of the three alanine linker residues were tested. Positive responses were only detectable for the linkers joining segments 4–5, 5–6, 8–9 and 12–13 (Additional file 1: Table S1). Importantly, flanking OLP harboring at least 8 residues before or after the AAA sequence also elicited a response in all but one of these cases, indicating that minor T cell reactivity to epitopes consisting of the full linker sequence was induced.

Finally, mice were assessed for the induction of humoral immune responses upon DNA.HTI vaccination and compared to animals vaccinated with full-length protein sequences. The ability to recognize full-length p55^gag^ and its processing products, the p37^gag^ intermediate (p17^gag^ plus p24^gag^) and p24^gag^, was assessed by Western blot assay using pooled plasma from immunized mice. Indeed, plasma from DNA.HTI vaccinated mice recognized the full-length and the processed Gag proteins (Figure 3F). Similar data were obtained with DNA.COMB vaccinated mice, although with a stronger signal indicating a higher antibody titer. Plasma samples were also analyzed by HIV-1 p24^gag^ ELISA and showed ~2 log lower antibody titers in HTI-immunized mice compared to mice vaccinated with DNA.COMB (Figure 3G). Thus, these two assays show that humoral responses induced by pDNA.HTI vaccination are able to recognize the mature proteins encoded by p55^gag^.

Together, these data demonstrate that despite lower *in vitro* accumulation in HEK293 cells, the HTI immunogen was able to induce robust and broadly distributed T-cell responses in DNA.HTI vaccinated mice, comparable in their magnitude to a mixture of plasmids expressing full-length proteins. In addition, the data show that the present HTI subunit design was able to prevent the strong Gag dominance that was induced by the full-length immunogen approach. The data also show that despite lower relative activity, HTI induces a humoral response that may serve as a tool to monitor vaccination outcome in future vaccine trials.

### MVA.HTI boost increases responses after DNA prime

**Figure 4:**
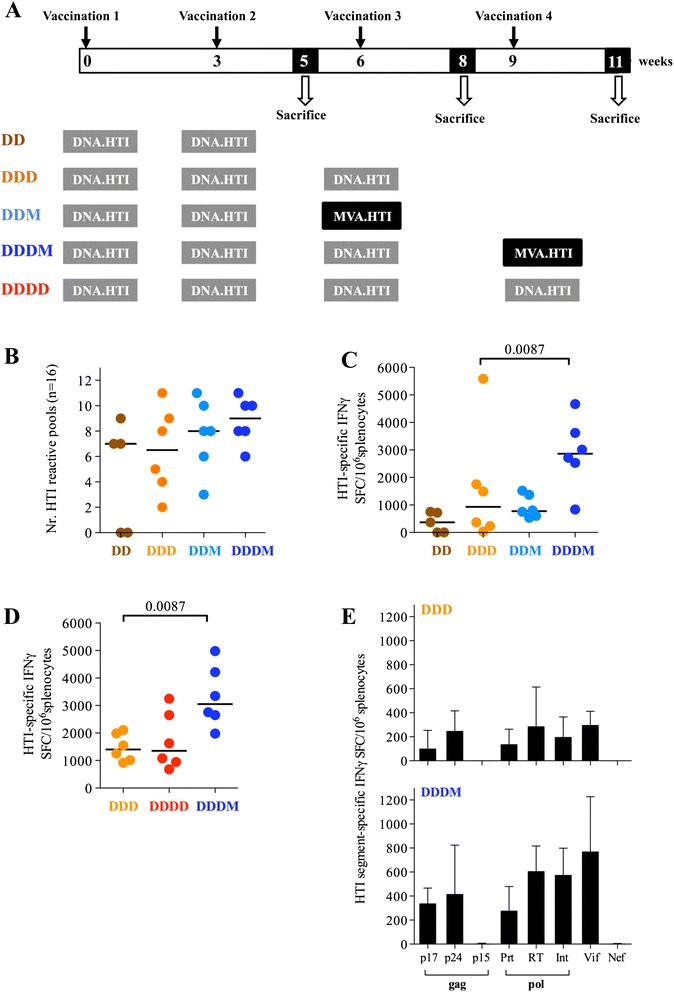
**HTI responses are boosted by MVA.HTI vaccination. (A)** Vaccination schedule of mice (n = 6/group) vaccinated with different prime/boost regimens including: 2xDNA (DD), 3xDNA (DDD), 4xDNA (DDDD); DNA prime followed by MVA boost (DDM and DDDM). 147 peptides (15-mers, overlapping by 11 residues) spanning the entire HTI sequence (including the leader sequence and linkers regions) were used in an IFN-γ ELISPOT assay to assess immunogenicity of the different regimens. Data from 1 animal in the DD group was not evaluated due to high background result in the ELISPOT assay. Breadth **(B)** and magnitude **(C-E)** of induced HTI-specific IFN-γ responses in different groups of mice to a set of 16 peptide pools spanning the different protein segments is shown either as total HTI-specific responses **(C, D)** or as response to individual proteins **(E)**. Bars represent median values. Median with interquartile range is shown in panel E. Panel D is an independent mouse experiment conducted to compare responses after DDD, DDDD and DDDM vaccination.

Poxvirus-vectors have shown great potential as HIV-1 vaccines and are an attractive choice to induce strong CD8^+^ T-cell responses [42,60,61]. MVA.HTI (M) was generated and used in C57BL/6 mice to compare different heterologous prime-boost combinations with DNA.HTI (D). Vaccines were delivered using 100 μg of DNA.HTI and 10^6^ pfu of MVA.HTI by IM injection (Figure 4A). Responses were measured 2 weeks after the last vaccination using pools of overlapping peptides (147 peptides, distributed in 16 linear pools spanning the HTI sequence, including the junctional regions).

DD immunization elicited responses in 3 out of 5 mice, whereas 100% of the animals showed responses after DDD vaccinations with a trend to increased levels compared to a DD regimen only (Figure 4B-C). A subsequent MVA.HTI boost after two or three DNA prime vaccinations led to higher responses with significantly higher magnitudes (median total magnitude of 777 vs 2,865 IFN-γ SFC/million splenocytes after DDD and DDDM, respectively, p = 0.0087; Figure 4C). To rule out that the boosted response observed was driven by the absolute number of immunizations rather than an heterologous prime-boost strategy, the immunogenicity of DDD, DDDD vs. DDDM vaccine regimens was assayed in an independent experiment. While a fourth DNA vaccination did not augment responses any further, a significant increase in the total magnitude of responses was found after a single MVA.HTI boost (Figure 4D), reaching in some animals more than 4,000 SFC/10^6^ spleen cells. As observed for the DDD only vaccinations, a similar balanced and broad response to most of the protein-subunits included in the immunogen was observed in all animals boosted with MVA.HTI (Figure 4E). Nef and Gag-p15 specific responses were only detected in 1 of 6 animals, possibly due to the short length of these two regions (13 and 27 amino acids, respectively; Table 3).

### HTI vaccination induces robust antigen-specific memory T cells in rhesus macaques

**Figure 5:**
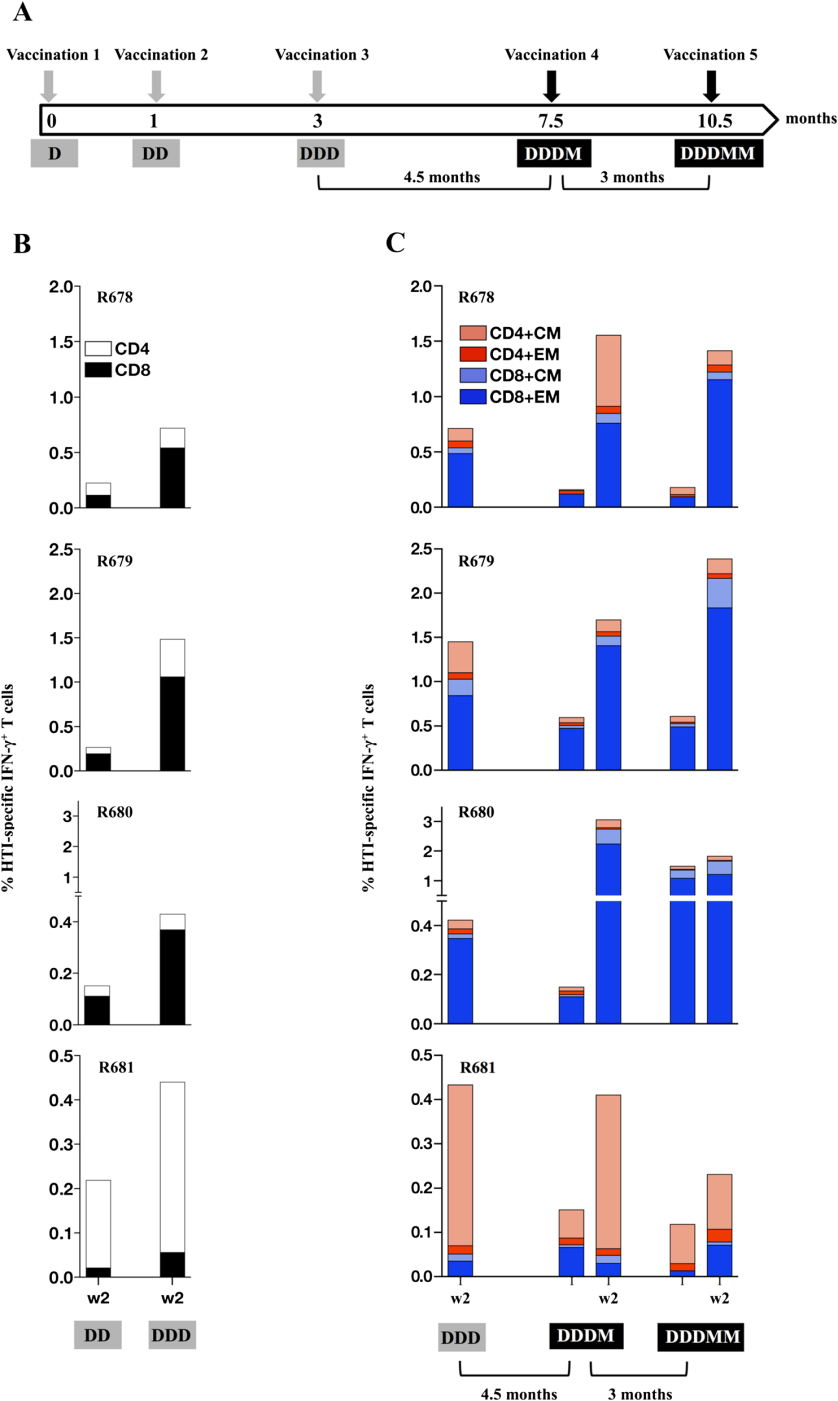
**HTI vaccine induces robust memory T cell responses in rhesus macaques. (A)** Vaccination schedule. Four macaques (R678, R679, R680, R681) were vaccinated 3x (0, 1, 3 months) with DNA.HTI and IL-12 DNA and subsequently with two MVA.HTI boosts. Cellular immune responses were measured by intracellular cytokine staining after the different vaccinations as indicated. **(B)** The frequency of HTI-specific IFN-γ^+^ T cells was measured after the DNA vaccinations using peptide pools covering the complete HTI sequence. The HTI-specific CD4^+^ (open bars) and CD8^+^ (filled bars) T cells are shown for each animal as number of reactive peptide pools. **(C)** The frequencies of HTI-specific IFN-γ^+^ T cells with central memory (CM; CD28^+^CD95^+^) and effector memory (EM; CD28^−^CD95^+^) phenotype are shown over the course of the study.

Next, four outbred Indian rhesus macaques with different MHC class I haplotypes (Table 6), none expressing Mamu-B*08 [62], were vaccinated via the IM route combined with *in vivo* electroporation at 0, 1 and 3 months with DNA.HTI (Figure 5A). A macaque IL-12 encoding DNA plasmid was co-administered as molecular adjuvant. The HTI-specific cellular immune responses were measured in PBMC upon stimulation with the immunogen-matched peptide pools and analyzed by intracellular cytokine staining. Analysis of the immune responses at 2 weeks after the 2^nd^ DNA vaccination showed robust induction of HTI-specific IFN-γ^+^ T cells in all macaques (Figure 5B). The responses increased with the 3^rd^ DNA vaccination yielding a range of 0.4-1.5% of total T cells reactive to HTI peptides.

**Table 6:** **Age, gender and haplotypes of macaques**

**Animal**	**Age (yrs)**	**Sex**	A*01	A*02	A*08	A*11	B*01	B*03	B*04	B*08	B*17
R678	3	Male	Neg	Neg	Pos	Neg	Neg	Neg	Pos	Neg	Pos
R679	3	Male	Neg	Neg	Neg	Neg	Neg	Neg	Neg	Neg	Neg
R680	3	Male	Neg	Neg	Neg	Neg	Neg	Neg	Neg	Neg	Neg
R681	3	Male	Neg	Neg	Pos	Neg	Neg	Neg	Neg	Neg	Neg

In three (R678, R679, R680) out of the four animals, the responses induced by HTI vaccination were mediated primarily by CD8^+^ effector memory T cells (EM, CD28^−^ CD95^+^) with significant contributions by the CD4^+^ and CD8^+^ central memory T cell subsets (CM, CD28^+^CD95^+^) (Figure 5C). In contrast, macaque R681 developed responses dominated by CD4^+^ T cells and, overall, had the lowest level of recognition of the HTI epitopes. Although we noted a contraction of the responses over the following 4.5 months, these responses persisted and were still in the range as measured after the DD vaccination (range 0.1-0.6% IFN-γ T cells), supporting the longevity of the DNA-vaccine induced immunity using an IM/EP approach [45,63]. At this time point, the animals were boosted with recombinant MVA.HTI (Figure 5A), which led to a 3- to 20-fold increase in the response, reaching 0.4-3.2% IFN-γ T cells (Figure 5C). In two of animals (R678, R680) the levels were higher than the peak response after three DNA immunizations. The responses persisted over the 3 months of follow-up and were successfully expanded in all 4 animals by a 2^nd^ MVA.HTI vaccination. Macaque R680 showed an exceptionally high persistence of responses (~2% of IFN-γ^+^ T cells), which were only weakly boosted by the 2^nd^ MVA boost. Overall, MVA.HTI was powerful in expanding DDD induced responses with limited benefit noted upon the 2^nd^ MVA.HTI boost.

### HTI vaccine induces antigen-specific memory T cells with cytotoxic potential

**Figure 6:**
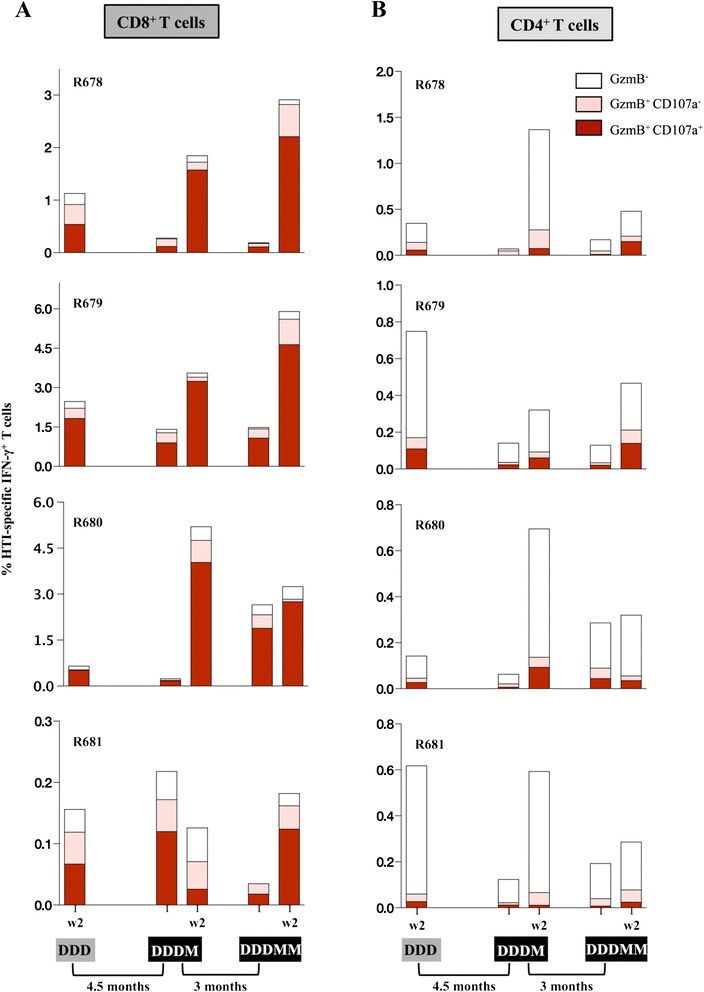
**HTI vaccine induces T cell responses with cytotoxic potential.** HTI-specific IFN-γ^+^ CD8^+^ **(A)** and CD4^+^ **(B)** subsets of T cells harboring granzyme B (GzmB) and/or expressing CD107a are shown for each animal.

We also analyzed the cytotoxic potential of the vaccine-induced T cell responses using Boolean gating strategies. Subsets of the antigen-specific CD8^+^ (Figure 6A) and CD4^+^ (Figure 6B) T cells were characterized by their granzyme B (GzmB) content and ability to degranulate, as determined by surface expression of CD107a after antigen stimulation [64]. This analysis revealed the majority of IFN-γ^+^ CD8^+^ T cells being double positive for CD107a^+^ and GzmB^+^ (Figure 6A), both after DNA prime and MVA boost vaccinations. Similar to the memory subsets, these cytotoxic phenotypes did not significantly change over time. The analysis of antigen-specific CD4^+^ T cells (Figure 6B) showed that although the majority of these cells were GzmB^−^ and CD107a^−^ , this subset also contained a small fraction of single CD107a^+^ or double positive (GzmB^+^ CD107^+^) cells. In this aspect the four animals showed a consistent pattern of effector functions, even though CD4^+^ T cells dominated the vaccine-induced response in animal R681.

### HTI vaccine induces broadly distributed responses in rhesus macaques

**Figure 7:**
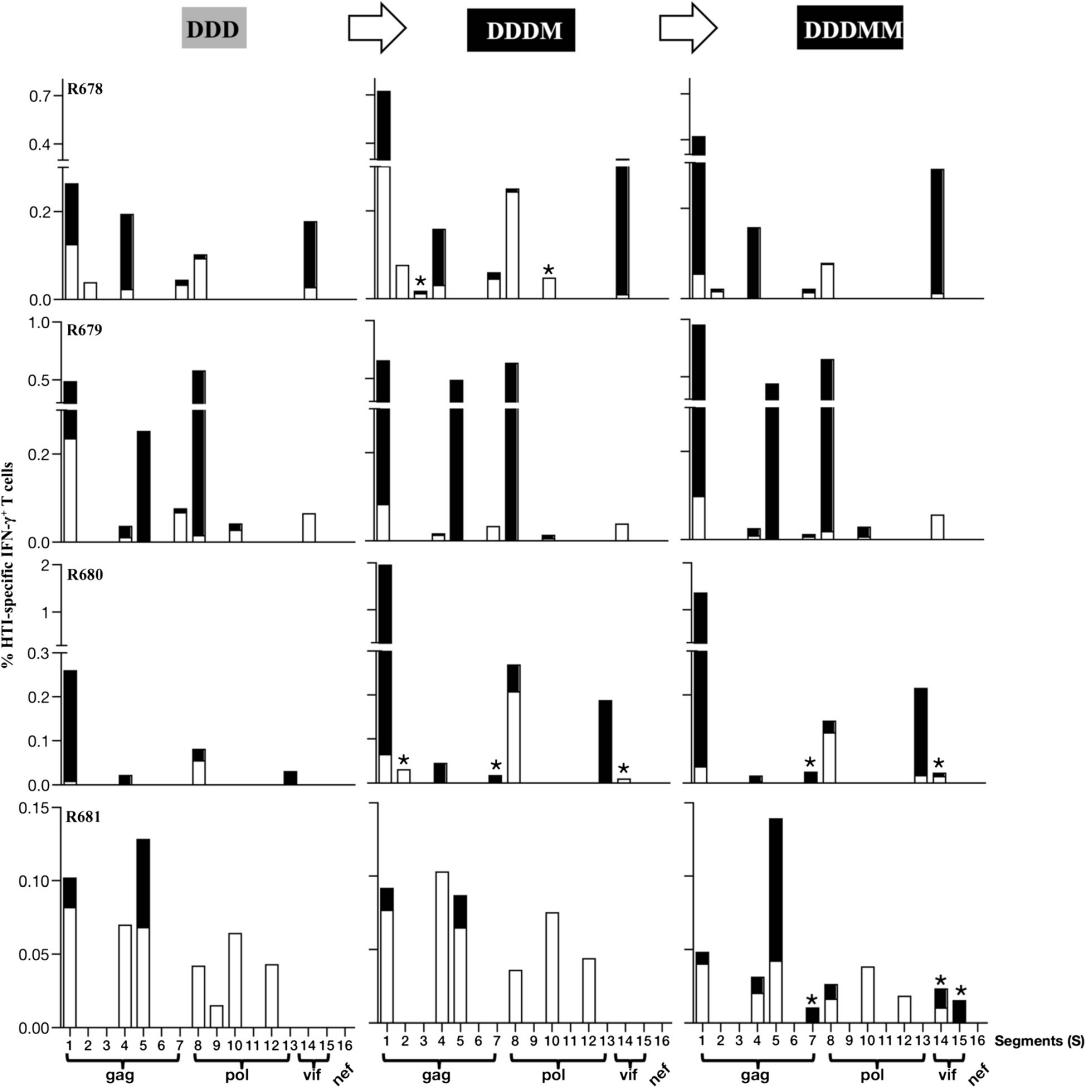
**Breadth of HTI-specific T cell responses in vaccinated macaques.** Mapping of HTI-specific T cell responses after the 3^rd^ DNA prime and 1^st^ and 2^nd^ MVA boost using peptide pools covering each segment (S1-S16). The percentage of IFN-γ^+^ CD4^+^ (open bars) and CD8^+^ (filled bars) T cells specific for each segment is shown. For a summary of data see Table 7. Asterisk denotes new responses emerging after MVA boost vaccination.

Finally, the distribution of responses across the different protein-subunits contained in the HTI immunogen was assessed at the peak after the DNA vaccinations (DDD) and after the MVA.HTI boosts (Figure 7). A set of 16 specific peptide pools (S1-S16), each covering a single segment of the HTI sequence (Table 3), was used to measure responses by flow cytometric intracellular cytokine staining assays. The results show that three DNA vaccinations induced T cell responses targeting all the proteins represented within HTI, except Nef and recognized a range of 4–7 of the 16 segments (Figure 7, Table 7). Of note, Nef is represented by a single segment of only 13 aa (and includes only three known CTL epitopes [55] restricted by the same B*07 supertype in humans), whereas other proteins were covered by several segments of various lengths (Table 7). Upon the 1^st^ MVA.HTI boost, the magnitude of the pre-existing responses was increased in all the animals (range 6–8 segments) and additional responses were found in two animals (R678, R680), albeit at low levels. After the 2^nd^ MVA.HTI boost, macaques R681 showed 3 additional responses, while some of the new responses found after the 1^st^ MVA.HTI boost in R678 and R680 were not detectable. Overall, DNA prime-MVA boost showed that 13 of the 16 segments of HTI were immunogenic in macaques (Table 7). As expected the majority of the HTI-specific responses was mediated by CD8^+^ IFN-γ^+^ T cells in R678, R679 and R680 and by CD4^+^ IFN-γ^+^ T cells in R681.

**Table 7:** **Breadth of cellular immune responses after DNA.HTI prime and MVA.HTI boost using HTI segment-specific peptide pools**

**Animal**	**Vaccine**	**Gag**							**Pol**						**Vif**		**Nef**	**# of positive segments**
		**p17**	**p24**					**p15**	**Prt**	**RT**			**Int**					
		**1** ^**a**^	**2**	**3**	**4**	**5**	**6**	**7**	**8**	**9**	**10**	**11**	**12**	**13**	**14**	**15**	**16**	
		**78** ^**b**^	**14**	**11**	**60**	**14**	**15**	**27**	**55**	**17**	**55**	**34**	**34**	**17**	**23**	**19**	**13**	
R678	DNA	+	+		+			+	+						+			6
	MVA	+	+	+^c^	+			+	+		+^c^				+			8
R679	DNA	+			+	+		+	+		+				+			7
	MVA	+			+	+		+	+		+				+			7
R680	DNA	+			+				+					+				4
	MVA	+	+^c^		+			+^d,e^	+					+	+^d,e^			7
R681	DNA	+			+	+			+	+	+		+					7
	MVA	+			+	+		+^d^	+		+		+		+^d^	+^d^		9

^a^segment number, S1-S16.

^b^number of amino acids per segment.

^c^new response detectable after 1^st^ MVA boost only.

^d^new response detectable after 2^nd^ MVA boost.

^e^new response detectable after 1^st^ and 2^nd^ MVA boost.

Because the HTI design is based on human, HLA-restricted T cell reactivity data, we did not further fine-map the HTI-segments to identify all CTL responses at the single epitope level in the context of each macaque MHC class I allele. Thus, the full breadth of the broad responses induced by HTI in macaques (81% of HTI segments) may be underestimated.

### HTI vaccine induces humoral responses in rhesus macaques

**Figure 8:**
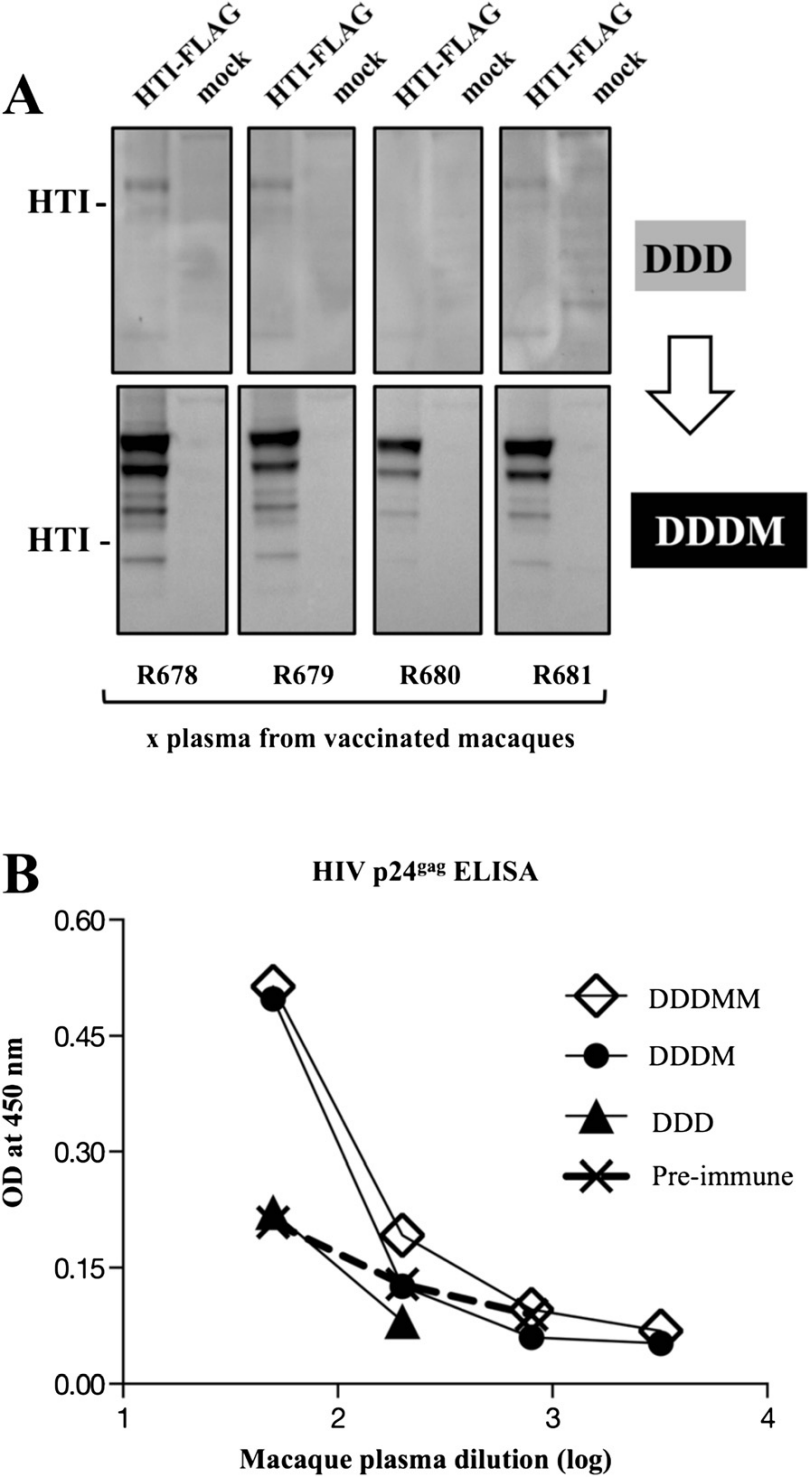
**Humoral responses in HTI vaccinated macaques. (A)** Binding antibodies to HTI were detected by Western immunoblot. The membranes contain enriched HTI-FLAG proteins from HEK293 cells. The membranes were probed with plasma (at a 1:100 dilution) from individual macaques collected at 2 weeks after 3rd DNA.HTI (DDD) and 1^st^ MVA.HTI (M) boost. **(B)** Anti-HIV-1 p24^gag^ antibodies were measured in different plasma samples, including pre-immune, after DNA.HTI and after MVA.HTI vaccinations of macaque R678 by a standard p24^gag^ ELISA. The graph shows absorbance (optical density, OD).

We also tested the plasma from the vaccinated macaques upon DNA prime and upon MVA boost for the development of humoral responses to HTI protein (Figure 8A). We found that upon 3 DNA vaccinations, 3 of the 4 animals (R678, R679, R801) developed low levels of antibodies specific for HTI protein. Upon MVA boost all 4 animals showed greatly increased responses. The plasma was further tested by ELISA for its ability to recognize p24^gag^. One of the macaques R678 also showed positive ELISA responses after the MVA boosts, indicating that the HTI vaccine sequence also induced humoral immune responses at least against some of its components.

Together, we present here the development and pre-clinical testing of a novel HIV-1 T-cell immunogen that has been rationally designed to maximize the induction of effective anti-viral T cell immunity. Its design is based on some of the largest available datasets of human cellular immunity to HIV-1, which allowed for the identification and inclusion of the most vulnerable T cell targets within the viral proteome that are associated with control of viremia. Thus, the HIVACAT T-cell Immunogen (HTI) differs conceptually from previous immunogen design approaches that have used full proteins sequences [23-25] or very short protein segments only [29,65,66]. This extends to the most promising approaches, including the one developed by Hanke et al. that focuses on conserved regions of the viral proteome [42]. In that design, some regions are included that do not seem to induce broad and strong immune reactivity in natural HIV-1 infection [36] and do not emerge as particularly beneficial targets [28]. Although the reasons for this reduced immunogenicity are unknown and could include subdominant beneficial targets [3], the relevance of such portions of vaccine inserts needs to be revisited. Similarly, immunogens that focus on highly conserved elements (CE) within Gag-p24 were chosen in part because of their preferential reactivity by controllers and since immune response to conserved regions have been associated with virologic control [17-20,28]. However, screening data showed that only select CE to be more frequently targeted by HIV controllers [27], in line with recent data suggesting that sequence conservation alone is not a reliable measure of vulnerability [67,68]. Finally, the favorable design of HTI is further supported by emerging data from studies in post-vaccination samples in the STEP and Phambili trials [69]. In those analyses, the subjects’ in vitro antiviral activity (measured by viral replication inhibition assay and associated with in vivo viral control [70] was directly correlated with the extent to which the CD8 T cell response was focused onto the HTI sequence, but not with the magnitude of responses to the rest of the viral proteome.

The incorporation of T cell reactivity data from natural HIV-1 infection may make the HTI sequence especially interesting for therapeutic and eradication approaches [71]. In this context, the inclusion of relatively conserved regions may help to cope with the consequences of viral evolution of both circulating and reactivated viruses [72] in chronically infected individuals. At the same time, the high density of potentially beneficial CD4^+^ T-cell epitopes may also help the induction of neutralizing humoral immunity in preventive settings [6,53]. In that setting, the focus on most vulnerable regions of the viral proteome could prove crucial if sterilizing immunity cannot be obtained as the induced T-cell responses may help to reduce viral fitness and lower viral set point in break-through infections. The induction of such effective anti-viral immunity, even though not sterilizing, would still have a far-reaching impact on the epidemic, both by reducing rate of disease progression as well as by lowering the likelihood of further virus transmission. Of note, and although the development of HTI was largely based on immune data from a HIV-1 clade B-infected cohort in Peru, our results are supported by additional analyses in an European clade B-infected cohort (shown here) and a US-based cohort previously described [36]. In addition, viral fitness data, which have been published since the initial design of this sequence, strongly support the HTI design and its preferential focus on sites that are highly susceptible to debilitating sequence mutations [56,68]. Furthermore, it is also reassuring that HTI in at least some regions overlaps with the HIVCons [42] the Conserved Elements [17,18] and other conserved regions previously proposed to induce protective T cell responses [31] despite the differences strategies that were the base for these designs.

An important aspect of the present vaccine candidate is its focus on fewer but possibly more effective targets than those that are generally induced by full-protein immunogens. Of particular importance may be the avoidance of responses to highly immunogenic and at the same time variable targets, as these may effectively misdirect the immune response [19]. This is especially the case for HIV-1 Nef, which contains multiple hot spots of high T-cell reactivity. In fact, past reports show that peptide sets spanning Nef contain the single most dominant 18-mer peptides that can elicit responses in more than 50% of the tested individuals [36]. This number is likely even an underestimation as responses to more variable regions are missed by the use of monomorphic peptide test sets [73,74]. The same may apply to responses targeting envelope which has shown surprisingly strong immunogenicity in some vaccination trials but which, in our analyses, did not yield any beneficial T cell targets [24,28,75]. The reactivity to potential decoy targets in envelope needs special consideration when using full-length envelope sequences for the induction of humoral responses. In this regard, shorter envelope immunogen designs (i.e. MPER [76]) may provide and advantage as there is less chance of diverting the T cell immune response to less effective epitopes.

The concern of decoy responses calls into question strategies that intend to overcome diversity by including multiple variants of the same sequence [32-35] as this may further prevent the induction of more beneficial T-cell specificities. As such, it may be beneficial to deal with HIV-1 sequence diversity by ignoring it, rather than trying to incorporating it into vaccine immunogens [77]. The expansion of responses to decoy targets has also important implications when considering HTI as a therapeutic vaccine. As suggested by previous therapeutic trials it may prove difficult to remodel the T cell response patterns induced during chronic HIV-1 infection by therapeutic vaccination [78-81]. In this case, the use of a vaccine vector with relatively low immunogenicity may help to specifically boost HTI-specific responses while avoiding the expansion of less beneficial T cell specificities that may occur when using viral vectors with more systemic immune activation. For this reason, we elected to express HTI first in a DNA plasmid vector as these allow for repeated administrations without generating vector immunity. In addition, DNA vaccines expressing different forms of simian immunodeficiency virus antigens decrease viremia upon SIVmac251 challenge [45,59].

Overall, murine data accurately predicted the outcome of vaccination in macaques, as in both species broadly directed responses of high magnitude were detected. Especially encouraging was the avoidance of overly immuno-dominant responses to single epitopes, reflected by the fact that in the macaque model 81% of the HTI-segments were recognized in the context of heterogeneous MHC haplotypes and after testing only four different animals. Why one animal failed to induce strong CD8 T cell responses and instead mounted broader CD4 T cell activities that were not boosted upon repeated MVA vaccination remains unclear. Further analyses in host genetics and/or immune homeostasis (cytokine levles, etc.) could possibly help explain this atypical response pattern and provide some leads to understand the only partial response rates even in the most potent vaccination strategies in SIV infected animals [82,83] Regardless of this, the avoidance of strong immunodominance effects will be a prerequisite for broad T cell responses *in vivo* a requirement that HTI, at least in mice and macaques, seems to fulfill as all components of the vaccine were evenly targeted. These observations need to be verified in human clinical trials as the antigen processing preferences and T cell receptor (TCR) repertoires in humans may be different. The same is true for the HTI-elicited high magnitude of antigen-specific effector memory CD8+ T cells with cytotoxic potential as well as CD4^+^ CM T cells. Hansen et al., using RhCMV vectored vaccines, reported that such effector-memory T cell responses located at the portal-of-entry are able to mediate viral control after SIV amplification in intra-rectally challenged rhesus macaques [83,84]. Of note, RhCMV vectored vaccines induced extremely broad responses with a significant altered immunodominance hierarchy compared to response to natural HIV infection, further suggesting that the fine specificity of the vaccine-induced HTI/HIV specific T cell response may prove crucial. Yet, since the HTI design is human data-driven, challenge studies in the macaque model are not really feasible as a) there are no comparable immune reactivity data from sufficiently large and out-bred monkey cohorts available and b) the evolution of SIV variants used in monkey models does not reflect the adaptation processes of HIV to the human population [85]. Thus, human clinical trials are being initiated in the therapeutic vaccination setting as a proof-of-concept to test whether this novel concept of HIV T cell immunogen design will indeed lead to the induction and refocus of broadly co-dominant T cell responses that can effectively suppress viral replication.

## Conclusions

We present here the design and preclinical-testing of a novel approach to HIV vaccine design, which is based on the identification of beneficial T cell responses and vulnerable viral targets and their subsequent inclusion in a refined subunit immunogen sequence. Our data show that the immunogen is capable to induce broadly and evenly distributed responses without being affected from strong immunodominance effects. Prime-boost strategies using DNA and MVA vectors elicit responses that exceed magnitudes by most other vaccine concepts and which are of a differentiation phenotype associated with vaccine-induced control of SIV infection in the macaque model. These data support the further development of this and other related functional-data based T cell vaccine approaches.

## Additional file

Additional file 1: Table S1. Fine-mapping of responses to linker-containing peptides in pDNA-HTI immunized mice.
