# Supplementary material for: A human immune data-informed vaccine concept elicits strong and broad T-cell specificities associated with HIV-1 control in mice and macaques
Source: J Transl Med. 2015 Feb 15;13:60. doi: 10.1186/s12967-015-0392-5 (PMC4336696; doi:10.1186/s12967-015-0392-5)
Supplement: Additional file 1: Table S1. — Fine-mapping of responses to linker-containing peptides in pDNA-HTI immunized mice. [file 12967_2015_392_MOESM1_ESM.pdf]

Supplementary Table 1. Fine-mapping of responses to linker-containing peptides in pDNA-HTI immunized mice[a].

HTI segment junctions	HIV protein	# of HTI OLP	Sequence of OLP	# of positive animals[a]
S1-S2	Gag p24	#27	QKIEVAAAKAFSPEV	
S2-S3	Gag p24	#31	PMFSALAAAGHQAAM	
S3-S4	Gag p24	#35	MQMLKEAAAIAPGQM	
S3-S4	Gag p24	#50	KIVRMYSPTSIAAAY	1
S4-S5[b]	Gag p24	#51	MYSPTSIAAAYVDRF	1
S4-S5	Gag p24	#52	TSIAAAYVDRFYKTL	1
S4-S5	Gag p24	#55	FYKTLRAEQAAA	1
S5-S6[b]	Gag p24	#56	YKTLRAEQAAACQGV	1
S5-S6	Gag p24	#57	RAEQAAACQGVGGPGH	
S6-S7	Gag p24	#61	ARVLAAACTERQANF	
S7-S8	Prt	#69	FLQSRAAAKMIGGI	
S7-S8[b]	Prt	#85	TQIGCTLNFAALVEI	2
S8-S9	RT	#86	CTLNFAALVEICTEM	2
S8-S9	RT	#87	FAALVEICTEMEK	
S9-S10	RT	#91	GKISKIAAALRWGF	
S10-S11	RT	#107	KLVGKLAAAILKEPV	
S10-S11	RT	#116	GQWTYQIYAAATKEL	
S11-S12	Int	#117	YQIYAAATKELQKQI	
S11-S12	Int	#125	PLWKGPAKLLWAAAK	
S11-S12	Int	#126	GPAKLLWAAAKIIR	2
S12-S13[b]	Int	#127	KLLWAAAKIIRDYGK	2
S12-S13	Int	#128	AAAKIIRDYGKQMA	1
S13-S14	Vif	#132	DCVAAAVKHHMYISK	
S14-S15	Vif	#139	STHPRAAAVTKL	
S15-S16	Vif	#145	PQKTKGHRAAAWLEA	

[a] Mice (N=6) were immunized 3 times with DNA.HTI and 4 of the animals with positive responses were used to fine-map the responses covering the linker-containing peptides.
[b]Alanine-linker including peptides that gave a positive response.
